# Integrated electronic prescribing and robotic dispensing: a case study

**DOI:** 10.1186/2193-1801-2-295

**Published:** 2013-07-02

**Authors:** Roderick J Beard, Peter Smith

**Affiliations:** Pharmacy Department, Sunderland Royal Hospital, Kayll Road, Sunderland, SR4 7TP UK; School of Pharmacy, University of Sunderland, Chester Road, Sunderland, SR1 3SD UK; Graduate research School, University of Sunderland, Chester Road, Sunderland, SR1 3SD UK

**Keywords:** Electronic prescribing, Automated dispensing robot, Benefits

## Abstract

**Introduction:**

To quantify the benefits of electronic prescribing directly linked to a robotic dispensing machine.

**Case description:**

Quantitative case study analysis is used on a single case. Hospital A (1,000 beds) has used an integrated electronic prescribing system for 10 years, and in 2009 linked two robotic dispensing machines to the system. The impact on dispensing error rates (quality) and efficiency (costs) were assessed.

**Evaluation and discussion:**

The implementation delivered staff efficiencies above expectation. For the out-patient department, this was 16% more than the business case had suggested. For the in-patients dispensary, four staff were released for re-deployment. Additionally, £500,000 in stockholding efficiency above that suggested by the business case was identified. Overall dispensing error rates were not adversely affected and products dispensed by the electronic prescribing - robot system produced zero dispensing errors. The speed of dispensing increased also, as the electronic prescribing - robot combination permitted almost instantaneous dispensing from the point of a doctor entering a prescription.

**Conclusion:**

It was significant that the combination of electronic prescribing and a robot eliminated dispensing errors. Any errors that did occur were not as a result of the electronic prescribing - robotic system (i.e. the product was not stocked within the robot). The direct linking of electronic prescribing and robots as a dispensing system together produces efficiencies and improves the quality of the dispensing process.

## Background

In 2005, the Department of Health in the UK issued a report authored by the Chief Pharmacist ‘Building a safer NHS for patients. –‘Improving medication safety’ (Smith, 2005). This paper discussed medication errors, their causes, and potential remedies, and built on previous work as reported in ‘An Organisation with a Memory: Building a safer NHS for Patients’ (Donaldson, 2001). The authors made many suggestions (Smith, 2005) for designing out errors through the use of a systems approach to medication systems. Electronic prescribing and robotic dispensing were put forward as potential tools to help reduce dispensing errors. However, the benefits that can be gained through the use of electronic prescribing and robots are not systematically documented in the literature, and it remains unclear as to what features provide the greatest safety and efficiencies. There is a variety of designs used in electronic prescribing and robotic dispensing systems, and it is also important when surveying the literature to consider the context of the medication system in a particular hospital. The Chief Pharmacist of England (Smith, 2005) quoted a study from the dispensing error analysis scheme [DEAS] published by Cardiff and Vale NHS trust. This paper analysed errors from 66 contributing hospitals between 1991 and 2001, covering 7000 errors. As such, it represents one of the biggest surveys of its kind in the UK. The errors as recorded by frequency are shown in Table 1. This illustrated the significance of prescribing error, and the potential for reduction.

**Table 1:** **Frequency and type of dispensing errors as reported by Smith** (2005)

Type of error	Proportion %
wrong drug supplied	23%
wrong strength of correct drug supplied	23%
wrong quantity	10%
wrong warnings or directions	10%
wrong drug name on the label	9%
wrong strength on label	8%
wrong form	7%
wrong patient name on label	7%

Beard (2009) described the benefits of electronic prescribing, and what features would contribute to those benefits. His study demonstrated that the greater the integration of electronic prescribing with other hospital systems, the greater the benefit. Similarly, over the last 10 years, robots have seen increasing use in hospitals, and whilst some of the benefits seem obvious, the precise features of a robot which yields the greatest benefit are yet to be identified. This paper uses case study analysis to explore the benefits which can be gained from linking electronic prescribing to a dispensing robot. The analysis is based on a single hospital, Hospital A.

### Aim of the study

The aim of this study is:

- To use case study analysis to analyse, explore and quantify the benefits of directly linking electronic prescribing and robotic dispensing in pharmacy.

The objectives are:

- To examine the model used in integrating electronic prescribing with a dispensing robot.
- To quantify the efficiencies that can be gained from the use of an integrated electronic prescribing – robot system.
- To quantify the efficiencies that can be gained from the use of an integrated electronic prescribing – robot system.

## Method

This study used case study analysis (Yin, 2011) to explore the benefits which can be gained from using an integrated electronic prescribing and robot system. The analysis is based on a single case of Hospital A. Hospital A has the following profile: a general population of 350,000; a sub-regional population of 750,000; 1,000 acute beds; 5,000 staff; and an income of £300 million. The hospital operates two dispensaries, including a smaller discrete out-patients pharmacy dispensing around 5,000 items per month.

The pharmacy within Hospital A has been operating an integrated electronic prescribing system for over 10 years (a Meditech system obtained from the USA). This system has modules for pharmacy, pathology, radiology, patient entry, electronic prescribing and medicine recording, and is the main recording system of the hospital. The pharmacy at Hospital A installed a robotic dispensing machine in 2009. The machine chosen was a triple-headed ROWA machine, with an automated labeller for each picking head. This machine stores products chaotically within itself, using product bar codes. A loading hopper and automatic loader for the machine was also purchased, which is around 10 metres long. The business case for the machine anticipated efficiencies, and it was estimated four whole time equivalent (WTE) technical staff would be released for re-deployment into other areas of the hospital. The pharmacy is open 80 hours a week and the staff savings were estimated across this time frame. Medication safety was also a feature of the business case. One crucial element of the project was the writing of the interface software that linked the unique product code in the Meditech electronic prescribing system to the ROWA robotic product system. The links had to be exclusive for each individual product, and it is this linkage between electronic prescribing and the robot, that yields the benefits. Within the business case, a smaller case for a second robot in the separate out–patient department was included. The service and software costs had already been considered for the main pharmacy robot, so the only additional cost was that of a single headed ROWA machine. This meant the business case for the out-patients pharmacy was smaller in anticipated efficiencies. The impact of linking technology on dispensing errors, staff efficiencies, and other efficiencies were assessed.

### The system

In this study we regard medication errors prevention in the same way as presented in the DEAS study (Smith, 2005), as shown in Table 2.

**Table 2:** **Types of errors prevented by electronic prescribing and robots** (**Smith,**^2005^)

Type of error	Proportion	Electronic prescribing prevents	Robot prevents	Electronic prescribing + Robot prevents
	Of errors			
wrong drug supplied	23%		Y	Y
wrong strength of correct drug supplied	23%		Y	Y
wrong quantity	10%		Y	Y
wrong warnings or directions	10%	Y		Y
wrong drug name on the label	9%	Y		Y
wrong strength on label	8%	Y		Y
wrong form	7%		Y	Y
wrong patient name on label	7%	Y		Y

It follows that provided electronic prescribing and robotic dispensing are integrated in a specific way; many dispensing errors can be ‘designed out ‘by skilful application of technology.

In the typical dispensing model, the process is as follows in the numbered sequence below:

1. Decision to discharge patient
2. Doctor writes prescription
3. Prescription delivered to pharmacy
4. Professional check of prescription
5. Prescription dispensed
6. Prescription checked
7. Prescription placed ready for delivery to ward

This process can take up to 4–8 hours for non-urgent items for a variety of reasons (Beard and Wood, 2010). The key point to remember is that in the pharmacy, the prescription and dispensed item can always be seen together until bagged for ward delivery.

**Figure 1:**
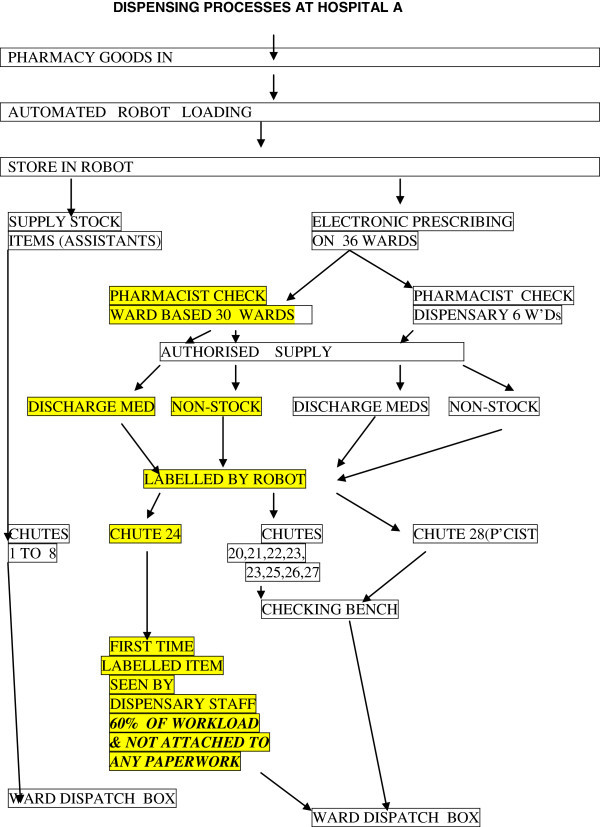
**Dispensing processes at hospital A.**

The process model used at Hospital A is shown in Figure 1.

## Results

**Figure 2:**
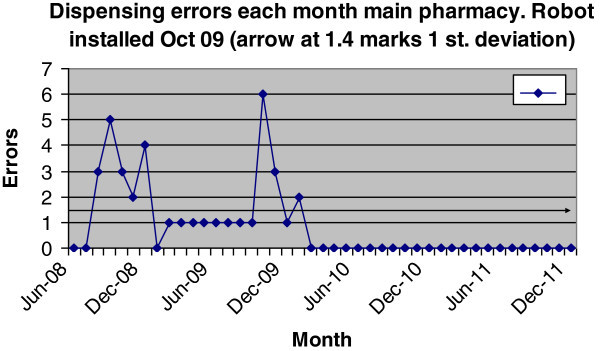
**Dispensing errors in in**-**patient pharmacy per month.** Electronic prescribing and the robot are linked after October 2009.

Dispensing errors per month were plotted on a control chart prior to, and after, the installation of the dispensing robot in October 2009. This is illustrated in Figure 2.

Control charts have been used in industry for many years as a means of assessing process control (Kelley, 1999). One difficulty in assessing processes with small numbers of deviations (e.g. dispensing errors) is their small number and the hap-hazard nature in which they occur. This may require assessing whether a small cluster of errors (deviations) is by chance, or whether there is something flawed within the process. Control charts are useful to help determine if it is more likely there is a systematic flaw. The technique basically examines the number of errors over a time frame, and calculates the standard deviation. Deviations above one standard deviation which consistently appear would suggest a more fundamental system flaw.

The out-patient pharmacy is open 45 hours a week, Monday to Friday. The business case for the outpatient robot required releasing for re-deployment around £70,000 in staff terms. On installing the robot, Band 5 technical staff could be replaced with lower banded dispensing staff, without adversely affecting the quality of the dispensing process. The change in skill mix was 50% (see Table 3). This was 16% more efficient than planned for in the business case. After installation, staff was reduced by 1.4WTE, and the skill mix was also adjusted to meet overall operational needs of the department. All NHS Hospitals in the UK pay staff on a banding system that equates all jobs to their value. The higher the job band, the more highly skilled the post. The job band and whole time equivalents for staff were determined, and used as a measure of the ‘quantity of skill’ to run the Outpatient pharmacy. The monetary value of the ‘skill quantity’ changes is calculated from the mid-point salary scale. The business case identified savings of around £35,000 per year for out-patients. The ability to reduce skill mix with regards to technicians gave an additional benefit.

**Table 3:** **Staff reduction by salary and grade after robot introduction in out**-**patient pharmacy**

Job band	WTE	Job band x WTE	Salary paid £	Midpoint salary £	New WTE	New skill amount	Salary total £
band 6	1	6	28000	28000	1	6	28000
band 6	1	6	28000	28000	1	6	28000
band 5	2.4	12	56040	23350	0	0	0
band 4	1	4	19500	19500	1	1	19500
band 3	1	3	17000	17000	1	1	17000
band 2	1	1	14360	14360	2	2	28720
totals	6.4	**32**	162900		5	**16**	121220
						**cost reduction**	**41680**
			WTE= whole time equivalent		skill reduction = 50%		
			BC = business case		BC =£35 k reduction		
					**Additional benefit over BC= 16%**		

The business case for the in-patient robot required four WTE staff to be released for other deployment to offset the £750,000 purchase costs. It was possible to release these staff, and deliver further economies. This was through a series of changes in working practices. Because the electronic prescribing - robot system only triggered after the pharmacist had professionally checked the prescription, and because the electronic links could not be interfered with, the previous “two dispensing checks” was deemed unnecessary. In effect, there was no need for higher skilled pharmacy technicians (Band 5) to be based in the dispensary, and so four were re-deployed to expand medicines management at ward level. This did not affect the 80 hours per week opening time for this dispensary. The dispensing and accuracy checking in the main pharmacy was mainly being done by Band 3 staff (dispensing assistants, not technicians). Technicians are used in a managerial capacity in the dispensary.

The dispensing processes, by being instantaneous, meant staff needed to walk about much less in the dispensary. The ROWA dispensing machine compacts around 3 kilometres of shelving into an area about 10 metres by 10 metres. Using automated loading meant staff time used to replenish shelves was significantly reduced. The discipline of having one location for product, and only being able to retrieve stock by using the standard processes, meant that inventory was reduced by 2 weeks, equating to around £500,000 in savings that *were additional to* the business case stock reduction of £250,000. Table 4 shows this.

**Table 4:** **Stock value** (£ **million**), **Stock issues** (£ **million**), **annual stock**-**turn and stockholding in weeks for the last 4 financial years at Hospital A**; **Note Year 08**/**09 is pre**-**robot**

		Stock value £	Issues £	Stockturn p/a	Stockholding in weeks
Year 08/09	Mean	1.69	1.22	8.60	6.05
Year 09/10	Mean	1.77	1.51	10.23	5.08
Year 10/11	Mean	1.62	1.70	12.58	4.13
Year 11/12	mean	1.74	1.99	13.72	3.79

Values in £'s millions.

## Discussion

The key findings of this study are that because electronic prescribing is integrated, when the doctor prescribes the medicine on the computer, he/she is also in fact writing the label to attach to the medicine. This means the label is *always* what the doctor requested. Because the label is always accurate to the prescription there can be no transcription error. Drugs can only be stored in the robot by bar code identification. There is a direct electronic link between the medicine, bar code, the item selected on the electronic prescription, and the label that the robot applies. These are the crucial links in deriving safety benefits from technology. To design in these links is to design out potential errors. Once designed, the system works from anywhere in the hospital. This allows 60% of dispensing activity to be triggered outside the pharmacy at Hospital A. Automatic labelling is a critical component of this system.

Another important consideration is avoidance wherever practically possible of part-packs. The robot does not handle these well in the way Hospital A operates the system, so avoidance of part packs is vital. Part packs cannot be entirely eradicated from use (e.g., steroids courses, chemotherapy), but minimising the number out of the robot is important. Once medication has been checked by a pharmacist (usually at ward level at Hospital A) the dispensing becomes nearly instantaneous. The remaining part of the process is to get the medication from pharmacy to the ward. In achieving instantaneous dispensing through the use of integrated electronic prescribing and a robot, the role of the dispensary pharmacist changes. No longer are pharmacists directly in control over the whole dispensing process. It is akin to craftsmen producing goods being replaced by production lines where quality control is through process control, and each individual is responsible for a part of the overall process, not all of it.

The prior use of electronic prescribing at Hospital A for 8 years meant that the integrated medicines management processes were well established. The delivery of products to the wards links in with these processes. Typically, most wards (30 out of 36) receive a medicines management service. Each ward can expect 0.75 WTE pharmacists, and 0.5 WTE technician time per week. Changes in skill-mix in out-patient department equates to an additional saving on top of staff reduction of 16%. Data from the control chart suggest de-skilling the dispensary workforce using robots has no impact on dispensing errors. Towards the end of 2009, there was an increase in dispensing errors, which was in part due to the consequences of the installation programme. This is where control charts proved useful to monitor the processes, especially when dealing with small numbers. A previous paper (Beard and Candlish, 2004) listed the different types of dispensing methods at Hospital A, and the error rates associated with them. Figure 2 shows a spike in errors just after installation. Error analysis showed them to be non-robot errors, i.e. they were picking errors from those shelves of the pharmacy where items cannot go into robots (part packs, round tubs of medicines, or items too small to be labelled by robot). Significantly, we have found zero errors for the robot plus electronic prescribing system combined, based on around 800,000 items per annum. This represents a huge benefit in safety. However, dispensing is not risk-free, since not all items are supplied and labelled from the robot. However, the opportunity for errors is significantly reduced.

The stock figures for 2008–09 represent values pre-robot. The business case required that besides re-deploying 4 staff, inventory value would be reduced by £250,000. This was achieved, but over time, the reduction in the number of weeks that stock is held has fallen by over 2 weeks, representing an additional saving of around £500,000 above the business case requirement. The cost of the robotic programme was around £750,000 over 10 years. This was achieved because the electronic prescribing - robot system allowed continuous reviewing of internal processes to yield better stock control.

Speed of turnaround time taken from the pharmacist’s clinical check is nearly instantaneous. At very busy periods dispensing times can rise to up to 20 to 30 minutes, but this situation tends not to last beyond about half an hour. Normally dispensing times, using traditional methods, can often be up to 4 hours for non-urgent dispensing (Beard and Wood, 2010). These authors quote how, by using lean processes, they reduced the dispensing time of a prescription from 4 hours to around 2 hours (these times include the time it takes a signed prescription to get from ward to pharmacy). This is not untypical of a traditional non-electronic prescribing – robotic system. The concept of instantaneous dispensing is not currently part of hospital pharmacy culture, nor is dispensing triggered from over 30 different points in the hospital.

Whittlesea et al. (2004) quotes a benchmark of 10 items per person per hour. The main Hospital A robot dispenses a maximum of 360 items per hour, equating to 36 dispensing staff. The in-patient pharmacy operates with around 10 dispensary staff. Hospital A’s robot chute 24 issues 60% of the dispensing activity, which is from the ward based pharmacy staff. This is not a directly comparable situation, but the efficiency is apparent.

## Conclusion

There are clear benefits in using electronic prescribing and robotic dispensing, and these will be realised so long as the following three conditions are met. The first is that the electronic prescribing system used is integrated with all the other hospital software systems (for transfer of information). The second is that the robotic dispenser is integrated to the electronic prescribing system, and the third is that there are automated labellers for those items robotically dispensed.

When the above conditions are applied several advantages become apparent, based on the principle of not needing to retype information. For items in the robot, there is no scope to make a dispensing error, improving patient safety; the process is much more efficient, and the skill mix of staff can be adjusted within the dispensary. As a consequence of all of the above, the speed of the prescription dispensing process increases dramatically. Another consequence of integrating electronic prescribing with a robot is that the purchasing of medicines needs to be given consideration so that items with appropriate bar codes are purchased, to ensure there is a direct link between product, robot and he electronic prescribing system. There is also a change in the professional model at Hospital A, as the dispensary pharmacist is no longer in complete control of the dispensing going on in the dispensary. This has wider implications for professional practice. The professional dispensing expertise is replaced to some extent by a systems approach. This allows flexibility in where dispensing is triggered (60% of the time at ward level by other pharmacists to dispensary pharmacist). It also allows pharmacists to maintain being at ward level rather than having to come back to the dispensary to help dispense. The ability to have ‘instantaneous dispensing’ means there is more time for the pharmacists to devote to the clinical care of the patient, and thereby doing more ‘value added’ clinical roles.
